# FKBP5 Induces Senescence in BMSCs and Inhibits Osteogenic Differentiation Through the Canonical WNT/β‐Catenin Signalling Pathway in Senile Osteoporosis

**DOI:** 10.1111/jcmm.70552

**Published:** 2025-04-20

**Authors:** Bin Zhu, Bowen Cai, Kaixiao Xue, Shumin Zhou, Guoyong Yin, Jiahu Fang

**Affiliations:** ^1^ Department of Orthopedics The First Affiliated Hospital with Nanjing Medical University Nanjing Jiangsu China; ^2^ Institute of Microsurgery on Extremities Shanghai Jiao Tong University Affiliated Sixth People's Hospital Shanghai China

**Keywords:** bone marrow stem cell, FKBP5, osteogenic differentiation, senile osteoporosis, WNT/β‐catenin signalling pathway

## Abstract

Senile osteoporosis and its associated fractures significantly contribute to increased morbidity, mortality, and healthcare costs among older adults. Further research is needed to elucidate the molecular mechanisms underlying senile osteoporosis. This study found that FKBP5 expression in bone marrow mesenchymal stem cells (BMSCs) increases with age and is inversely correlated with patients' bone mineral density and CT values. Functional analyses revealed that FKBP5 plays a crucial regulatory role in BMSC osteogenic differentiation, acting through the canonical WNT/β‐catenin signalling pathway. FKBP5 binds to β‐catenin, promoting its ubiquitination and degradation. Importantly, administration of SAFit2, a selective FKBP5 inhibitor, enhanced bone mineral density in an animal model of senile osteoporosis. These findings suggest that FKBP5 may represent a novel therapeutic target and provide new insights into the treatment of senile osteoporosis.

## 1 Introduction

Osteoporosis is a metabolic, degenerative condition characterised by weakened bone structure and reduced bone mass [1, 2]. The leading causes are oestrogen deficiency and ageing, with the age‐related form known as senile osteoporosis [3]. This typically occurs after age 70 and is marked by a decline in bone mineral density [2, 4]. Senile osteoporosis and associated fractures exert profound impacts on the health and life quality of older adults, while also imposing substantial burdens on public healthcare systems [5]. However, effective therapeutic and prevention strategies remain limited due to incompletely elucidated molecular mechanisms underlying senile osteoporosis [6, 7, 8]. Further research is needed to explore the molecular underpinnings of this condition.

Deficits in bone formation constitute a primary driver of age‐related bone loss [9]. This can occur through two mechanisms: extrinsic alterations to the bone microenvironment mediated by growth factors and hormones, and intrinsic senescence of bone marrow‐derived mesenchymal stem cells (BMSCs), which impairs osteoblastic differentiation and the number of osteoblasts [10, 11]. Studies indicate that the ageing process significantly disrupts the inherent characteristics of BMSCs, including their propensity for senescence and ability to differentiate into osteogenic and adipogenic lineages. Maintaining equilibrium between these differentiation pathways is crucial for preserving bone homeostasis, and BMSC senescence represents a critical factor in the development and progression of osteoporosis. Enhancing the osteogenic differentiation and efficiency of BMSCs is a promising area of research for treating senile osteoporosis.

FK506 binding proteins (FKBPs) are immunophilins that interact with steroid receptors and molecular chaperones such as heat shock proteins [12]. FKBP5 acts as a negative regulator of the glucocorticoid receptor, attenuating its transcriptional activity [13]. Previous studies have shown that FKBP5 is abundantly expressed in the central nervous system (CNS) and modulates the mTOR pathway, thereby influencing neuronal development, protein folding, and hormonal regulation [14, 15]. Notably, FKBP5 has been implicated in neurological disorders, including post‐traumatic stress disorder, Alzheimer's disease, Parkinson's disease, and schizophrenia [14, 16, 17, 18]. Additionally, FKBP5 has been reported to contribute to ageing‐related phenotypes, such as elevated cardiovascular risk and heightened inflammation [19, 20]. However, the precise contributions and mechanisms of FKBP5 in senile osteoporosis remain unknown. This study aims to investigate whether FKBP5 modulates BMSC osteogenic differentiation and its potential role in senile osteoporosis pathogenesis.

## 2 Results

### 2.1 FKBP5 Expression in BMSCs From Elderly Donors Is Elevated

**FIGURE 1:**
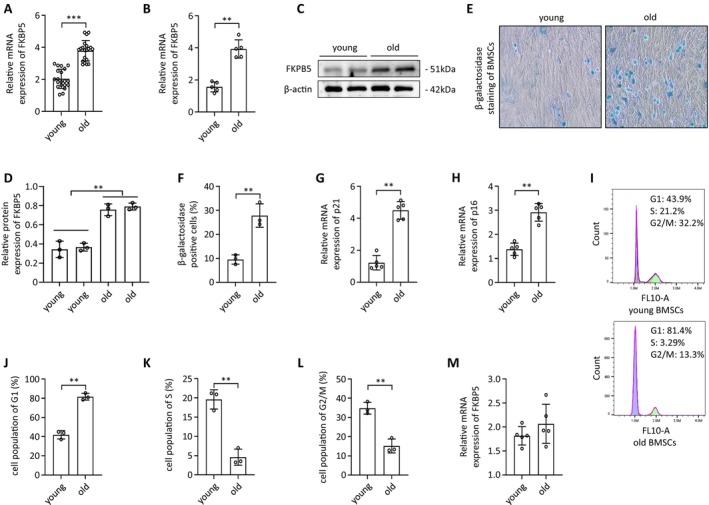
FKBP5 Expression in BMSCs from Elderly Donors is Elevated. (A) FKBP5 expression in bone tissues of the young and old donors; (B–D) FBKP5 expression in mRNA and protein levels in BMSCs from young and old donors; (E, F) β‐galactosidase staining in BMSCs from the young and old donors; (G, H) The expression of senescence‐related genes in BMSCs from the young and old donors; (I–L) Cell cycle analysis of BMSCs from young and old doners; (M) FKBP5 expression in osteoclasts from young and old donors.

To investigate the role of FKBP5 in ageing and osteoporosis, we first assessed its expression in bone tissues from young and older donors, with young donors aged 18–37 years and older donors aged 66–93 years. Our findings revealed significantly elevated FKBP5 expression in the bone tissue of older donors (Figure 1A). Bone tissue is a complex structure composed of various cell types, including BMSCs, osteocytes, osteoclasts, and osteoblasts, as well as supporting haematopoietic cells. Different cell types likely contribute to the increased FKBP5 expression observed. As our study focused on the mechanisms of osteoclast and osteoblast activity in bone remodelling, we isolated BMSCs from both age groups. Quantitative real‐time PCR (qRT‐PCR) and western blotting confirmed elevated levels of FKBP5 mRNA and protein in BMSCs from older donors (Figure 1B–D). Moreover, the positivity rate for β‐galactosidase staining, a marker of cellular senescence, was notably higher in the senior group (Figure 1E,F). Additionally, the senescence markers p21 and p16 were expressed at significantly higher levels in the older BMSCs (Figure 1G,H). Cell cycle analysis demonstrated that aged BMSCs were primarily arrested in the G1 phase, indicating reduced proliferation capability (Figure 1I–L). In contrast, no significant differences in FKBP5 expression were detected in osteoclasts between young and older donors (Figure 1M). These data confirm that FKBP5 expression is upregulated in BMSCs with ageing and further validate the senescent phenotype of older BMSCs.

### 2.2 FKBP5 Is a Key Regulator of Osteogenic Differentiation and Senescence

**FIGURE 2:**
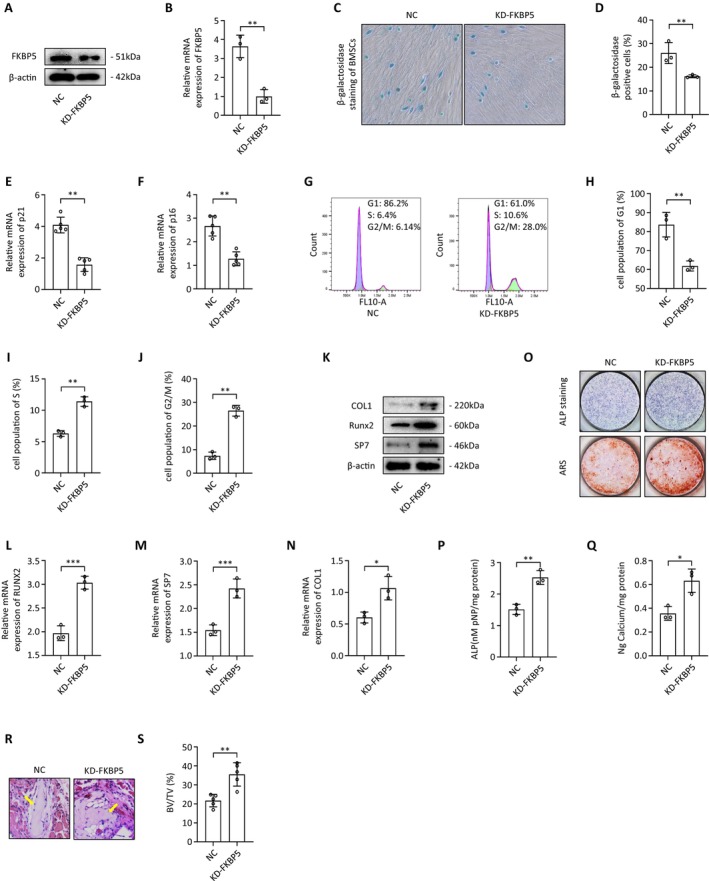
FKBP5 downregulation enhanced osteogenic differentiation and reduced senescence. (A, B) The efficacy of FKBP5 specific shRNA was measured by qRT‐PCR and western blotting; (C, D) β‐galactosidase staining in BMSCs from NC and KD‐FKBP5 groups; (E, F) The expression of senescence‐related genes in BMSCs from NC and KD‐FKBP5 groups; (G–J) Cell cycle analysis of BMSCs from NC and KD‐FKBP5 groups; (K–N) The expression of osteogenic markers was evaluated at the mRNA and protein levels; (O, P) ALP staining was performed after BMSCs were exposed to an osteogenic induction medium for 7 days; (O, Q) Alizarin Red staining was performed after BMSCs were exposed to an osteogenic induction medium for 14 days; (R) Representative H&E staining images of transplants from NC and KD‐FKBP5 groups (yellow arrows represent newly formed bone); (S) Quantitative assessment of the bone tissue of transplants from NC and KD‐FKBP5 groups.

To clarify FKBP5's role in the osteogenic differentiation of BMSCs, we employed FKBP5‐specific shRNA to inhibit its expression, assessing the knockdown efficiency through qRT‐PCR and western blotting (Figure 2A,B). We observed that the positivity rate for β‐galactosidase staining was substantially lower in the FKBP5 downregulated group (Figure 2C,D). Furthermore, the senescence markers p21 and p16 showed significantly lower expression levels (Figure 2E,F). Cell cycle analysis indicated that BMSCs with diminished FKBP5 expression exhibited less G1 phase arrest, suggesting reduced senescence (Figure 2G–J). After seven days of osteogenic induction medium treatment, we evaluated the expression of osteogenic markers. Notably, RUNX2 (Runt‐related transcription factor 2), SP7 (Transcription Factor Sp7), and COL1A1 (Collagen, Type I, Alpha 1) expressions increased at both mRNA and protein levels following FKBP5 knockdown (Figure 2K–N). Alkaline phosphatase (ALP) and Alizarin Red staining conducted after exposure to the osteogenic induction medium for 7 and 14 days demonstrated increased ALP activity and elevated extracellular matrix mineralisation in BMSCs with reduced FKBP5 expression (Figure 2O–Q).

**FIGURE 3:**
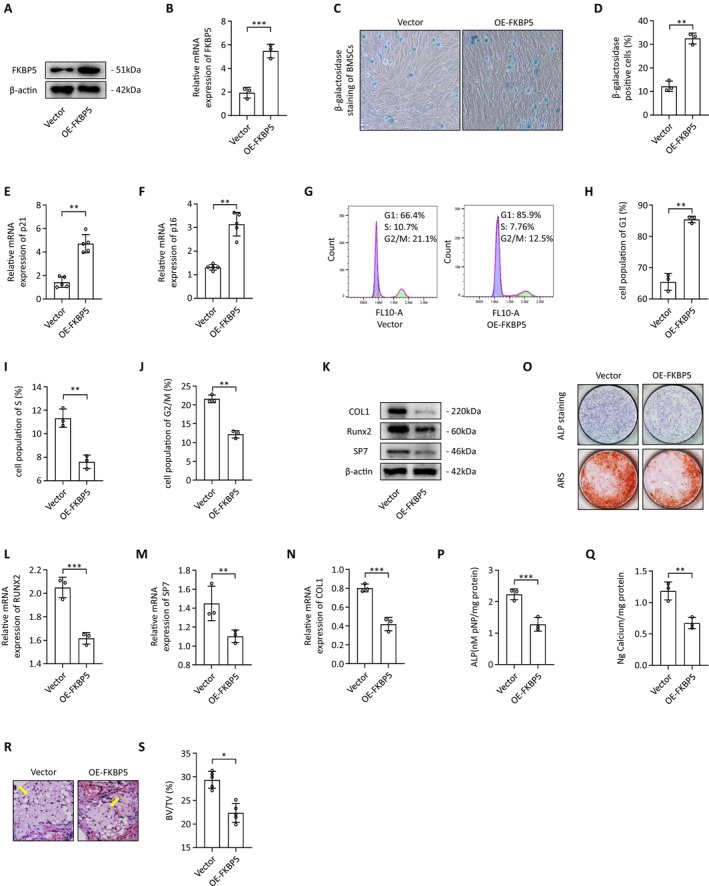
FKBP5 upregulation reduced osteogenic differentiation and enhanced senescence. (A, B) The efficacy of FKBP5 specific lentivirus was measured by qRT‐PCR and western blotting; (C, D) β‐galactosidase staining in BMSCs from Vector and OE‐FKBP5 groups; (E, F) The expression of senescence‐related genes in BMSCs from Vector and OE‐FKBP5 groups; (G–J) Cell cycle analysis of BMSCs from Vector and OE‐FKBP5 groups; (K–N) The expression of osteogenic markers was evaluated at the mRNA and protein levels; (O, P) ALP staining was performed after BMSCs were exposed to an osteogenic induction medium for 7 days; (O, Q) Alizarin Red staining was performed after BMSCs were exposed to an osteogenic induction medium for 14 days; (R) Representative H&E staining images of transplants from Vector and OE‐FKBP5 groups (yellow arrows represent newly formed bone); (S) Quantitative assessment of the bone tissue of transplants from Vector and OE‐FKBP5 groups.

To further delineate the role of FKBP5 in osteogenic differentiation, we used an FKBP5‐specific lentivirus to enhance its expression in BMSCs and evaluated the overexpression efficiency via qRT‐PCR and western blotting (Figure 3A,B). Conversely, the β‐galactosidase staining positivity rate was notably higher in the FKBP5 upregulated group (Figure 3C,D). Moreover, the senescence markers p21 and p16 were expressed at significantly increased levels (Figure 3E,F). Cell cycle analysis indicated that BMSCs with upregulated FKBP5 expression were arrested in the G1 phase, reflecting impaired proliferation (Figure 3G–J). The expression levels of osteogenic markers, including RUNX2, SP7, and COL1A1, were significantly inhibited in conjunction with elevated FKBP5 expression (Figure 3K–N). ALP and alizarin red staining confirmed that increased FKBP5 expression notably decreased ALP activity and inhibited extracellular matrix mineralisation and calcium nodule formation (Figure 3O–Q).

To validate our in vitro findings, we examined the effects of FKBP5 on bone formation in vivo. BMSCs were incubated with β‐TCP (tricalcium phosphate) and subsequently transplanted into the muscle pockets of immunocompromised eight‐week‐old mice. Histological analysis conducted eight weeks post‐transplantation showed that BMSCs with lower FKBP5 expression formed more bone structures compared to the control group (Figure 2R), while BMSCs with higher FKBP5 expression yielded fewer bone structures (Figure 3R). Quantitative assessments revealed statistically significant differences in bone tissue formation between the groups (Figures 2S and 3S).

### 2.3 Association of FKBP5 Expression With Senile Osteoporosis

**FIGURE 4:**
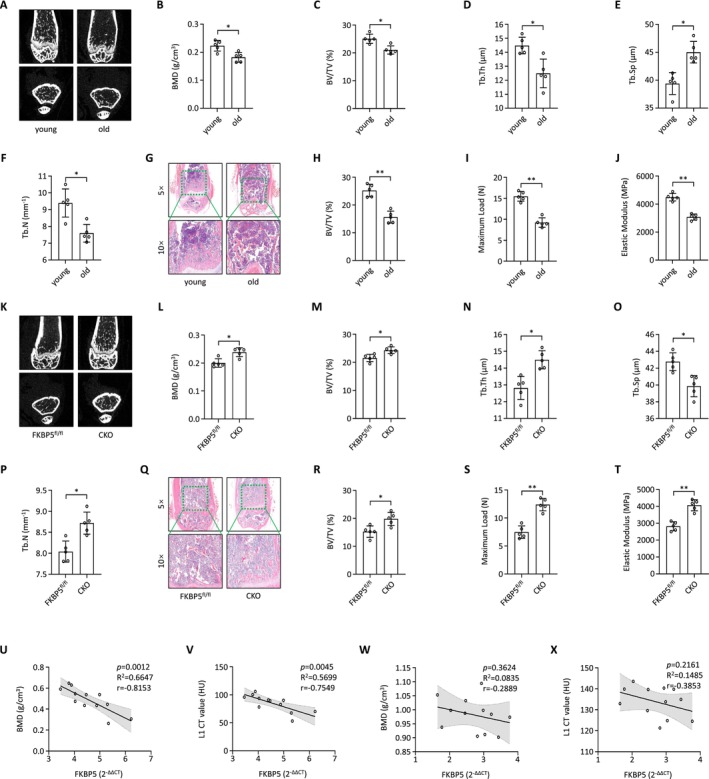
FKBP5 expression is closely correlated with senile osteoporosis in vivo and in the clinic. (A) Represent micro‐CT images showed bone mass and microstructures of the young and the senile osteoporosis mice; (B–F) Measurement of bone mineral density (BMD), trabecular separation (Tb. Sp), bone volume/total volume (BV/TV), trabecular thickness (Tb. Th), and trabecular number (Tb. N) of femurs in the young and the senile osteoporosis mice; (G, H) Representative H&E staining images and quantitative assessment of femurs in the young and the senile osteoporosis mice; (I, J) The biomechanical of femur bone strength was performed using three‐point bending test and reflected as elastic modulus (N) and maximal load (MPa); (K) Represent micro‐CT images showed bone mass and microstructures of FKBP5 CKO mice and FKBP5^fl/fl^ mice; (L–P) Measurement of bone mineral density (BMD), trabecular separation (Tb. Sp), bone volume/total volume (BV/TV), trabecular thickness (Tb. Th), and trabecular number (Tb. N) of femurs in the FKBP5 CKO mice and FKBP5^fl/fl^ mice; (Q, R) Representative H&E staining images and quantitative assessment of femurs in the FKBP5 CKO mice and FKBP5^fl/fl^ mice; (S, T) The biomechanical of femur bone strength was performed using three‐point bending test and reflected as elastic modulus (N) and maximal load (MPa); (U) Correlation analysis of FKBP5 expression and bone mineral density (BMD) of L1 lumbar in osteoporosis patients; (V) Correlation analysis of FKBP5 expression and CT value of L1 lumbar in osteoporosis patients; (W) Correlation analysis of FKBP5 expression and bone mineral density (BMD) of L1 lumbar in age‐paired healthy donors; (X) Correlation analysis of FKBP5 expression and CT value of L1 lumbar in age‐paired healthy donors.

To further examine the role of FKBP5 in senile osteoporosis, a model was established using 12‐month‐old mice (Baseline data in Table S1). Micro‐CT analysis revealed significant bone marrow loss in the femur, as indicated by reductions in bone mineral density (BMD), trabecular separation (Tb.Sp), bone volume/total volume (BV/TV), trabecular thickness (Tb.Th), and trabecular number (Tb.N) (Figure 4A–F). Additionally, H&E (Haematoxylin and eosin) staining corroborated the loss of femoral bone marrow in aged mice (Figure 4G,H). Biomechanical testing demonstrated that both the elastic modulus and maximal load of the senile mice were lower than those of their younger counterparts (Figure 4I,J). To explore the relationship between FKBP5 and senile osteoporosis, we specifically deleted FKBP5 in BMSCs by interbreeding FKBP5^flox/flox^ mice with Prx1‐cre transgenic mice, resulting in FKBP5 conditional knockout (FKBP5 CKO) mice. Micro‐CT analysis indicated that cancellous trabecular architecture was better preserved in 12‐month‐old male FKBP5 CKO mice compared to FKBP5^fl/fl^ mice (Figure 4K–P), as reflected in BMD, Tb.Sp, BV/TV, Tb.Th, and Tb.N metrics. H&E staining further illustrated the preservation of femoral cancellous trabecular structures in FKBP5 CKO mice (Figure 4Q,R). Importantly, FKBP5 CKO mice exhibited significantly higher elastic modulus and maximal load compared to FKBP5^fl/fl^ mice in biomechanical testing (Figure 4S,T).

We also assessed FKBP5 expression in BMSCs from osteoporosis patients versus age‐matched healthy controls. The results showed a significant inverse correlation between FKBP5 expression and both BMD and CT values for the L1 lumbar spine in osteoporosis patients (Figure 4U,V). In healthy donors, however, no correlation was observed between FKBP5 expression and either BMD or CT values of the L1 lumbar vertebrae (Figure 4W,X). These findings indicate a strong association between FKBP5 expression and senile osteoporosis both in vivo and clinically.

### 2.4 Administration of SAFit2 Alleviates Senile Osteoporosis

**FIGURE 5:**
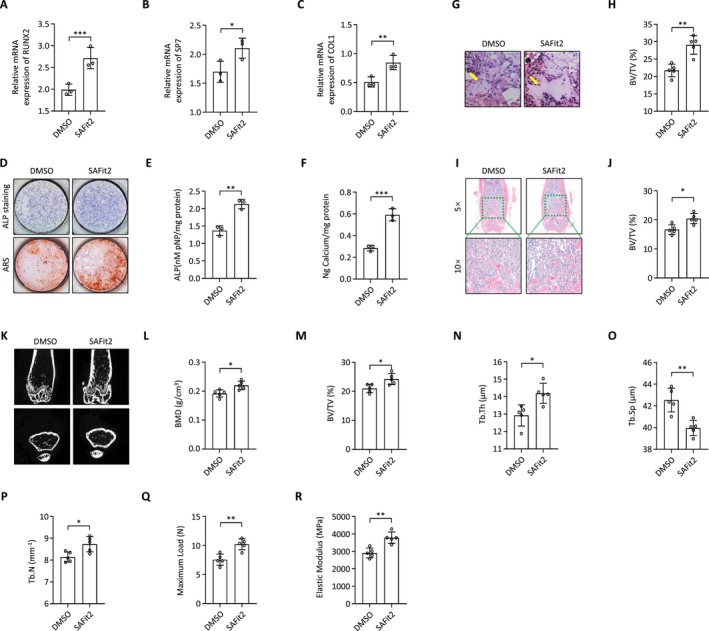
Administration of SAFit2 alleviates senile osteoporosis. (A–C) The expression of osteogenic markers was evaluated at the mRNA level after 48 h of exposure to SAFit2 with a density of 500 nM; (D–F) ALP and alizarin red staining were performed on days 7 and 14 of osteogenic induction medium treatment; (G, H) Representative H&E staining images and quantitative assessment of transplants from SAFit2 pretreated group and the control group (yellow arrows represent newly formed bone); (I, J) Representative H&E staining images and quantitative assessment of femurs in SAFit2 treated group and the control group; (K) Represent micro‐CT images showed bone mass and microstructures of femurs in SAFit2 treated group and the control group; (L–P) Measurement of bone mineral density (BMD), trabecular separation (Tb. Sp), bone volume/total volume (BV/TV), trabecular thickness (Tb. Th), and trabecular number (Tb. N) of femurs in SAFit2 treated group and the control group; (Q, R) The biomechanical of femur bone strength was performed using three‐point bending test and reflected as elastic modulus (N) and maximal load (MPa).

We initially assessed the effect of SAFit2, a selective inhibitor of FKBP5, on the osteogenic differentiation of BMSCs. Following 48 h of SAFit2 exposure at a concentration of 500 nM, cells were treated with osteogenic induction medium for seven days, and osteogenic marker levels were measured. RUNX2, SP7, and COL1A1 expressions increased significantly at both mRNA and protein levels in the SAFit2‐treated group (Figure 5A–C). ALP and alizarin red staining performed on days 7 and 14 during osteogenic induction demonstrated that SAFit2 treatment markedly enhanced BMSC osteogenic differentiation, as indicated by increased extracellular matrix mineralisation and ALP activity (Figure 5D–F). Muscle pocket experiments confirmed SAFit2's role in promoting osteogenic differentiation, showing that SAFit2‐pretreated BMSCs formed more bone tissue compared to the control group (Figure 5G,H). Subsequent investigations revealed that daily intraperitoneal treatment with SAFit2 at a dose of 20 mg/kg resulted in reduced bone loss (Figure 5K–P), as reflected through BMD, Tb.Sp, BV/TV, Tb.Th, and Tb.N. H&E staining of the distal femur corroborated the reduction in bone loss (Figure 5I,J). Furthermore, the three‐point bending test indicated a significantly higher maximum load and elastic modulus in the SAFit2‐treated group compared to controls (Figure 5O,P).

### 2.5 FKBP5 Modulation Impacts BMSC Osteogenic Differentiation via the Canonical Wnt Signalling Pathway

**FIGURE 6:**
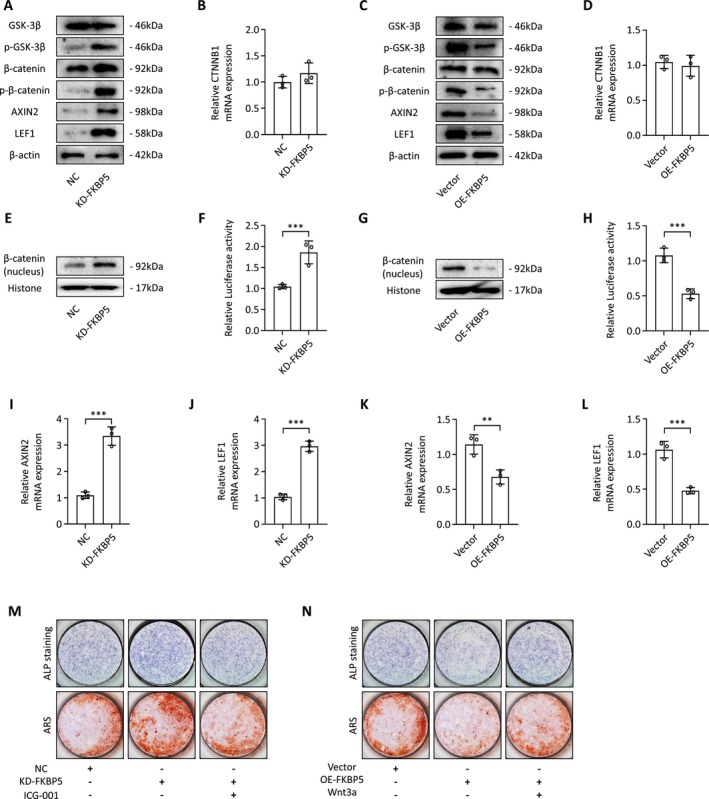
FKBP5 Modulation Impacts BMSC Osteogenic Differentiation via the Canonical Wnt Signalling Pathway. (A, C) The expression of key proteins in canonical Wnt signalling pathway were measured using western blotting; (B, D) The expression of β‐catenin was measured using qRT‐PCR; (E, G) The expression of nucleus β‐catenin was measured using qRT‐PCR; (F, H) TOPFlash reporter assay was performed to identify the effect of FKBP5 on canonical Wnt signalling pathway; (I–L) The mRNA expression of canonical Wnt signalling target genes were measured using qRT‐PCR; (M) ALP staining and AR staining showed that ICG‐001 inhibited the increased osteogenic potential of BMSCs induced by the downregulation of FKBP5 expression; (N) ALP staining and AR staining showed that Wnt3a restored the reduced ability of BMSCs to produce bone owing to the upregulation of FKBP5 expression.

Canonical Wnt signalling plays a crucial role in both bone homeostasis and the differentiation of BMSCs into osteogenic cells. We first examined β‐catenin expression to determine whether FKBP5 influences osteogenesis through Wnt signalling. Our results showed that downregulating FKBP5 expression resulted in elevated β‐catenin protein levels while upregulating FKBP5 expression led to reduced β‐catenin levels (Figure 6A,C). However, qRT‐PCR did not reveal any significant changes in β‐catenin mRNA levels in relation to FKBP5 expression (Figure 6B,D). Additionally, FKBP5 downregulation was associated with increased phosphorylation of β‐catenin, whereas FKBP5 overexpression decreased this phosphorylation (Figure 6A,C). The downregulation of FKBP5 also promoted the nuclear accumulation of β‐catenin, while its upregulation inhibited this effect (Figure 6E,G). We assessed the phosphorylation of GSK‐3 at Ser9, a critical step in Wnt signalling activation. Although overall GSK‐3 levels remained stable, phosphorylated GSK‐3 at Ser9 was increased with FKBP5 downregulation and decreased with FKBP5 upregulation (Figure 6A,C). Luciferase activity assays demonstrated that FKBP5 upregulation suppressed TOPFlash reporter activity, while its downregulation increased activity (Figure 6F,H). Additionally, we investigated FKBP5's regulatory effects on AXIN2 and LEF1, known targets of canonical Wnt signalling. We found that the mRNA expressions of these genes were decreased when FKBP5 was upregulated and increased upon downregulation (Figure 6I–L). These results were confirmed at the protein level using western blotting (Figure 6A,C). Notably, treatment with ICG‐001, a Wnt signalling inhibitor, decreased the osteogenic potential of BMSCs induced by FKBP5 downregulation, as shown by ALP and AR staining (Figure 6M). Conversely, treatment with Wnt3a, a Wnt signalling activator, restored the osteogenic capacity of BMSCs that had undergone FKBP5 upregulation (Figure 6N).

### 2.6 FKBP5 Is Essential for β‐Catenin Stabilisation

**FIGURE 7:**
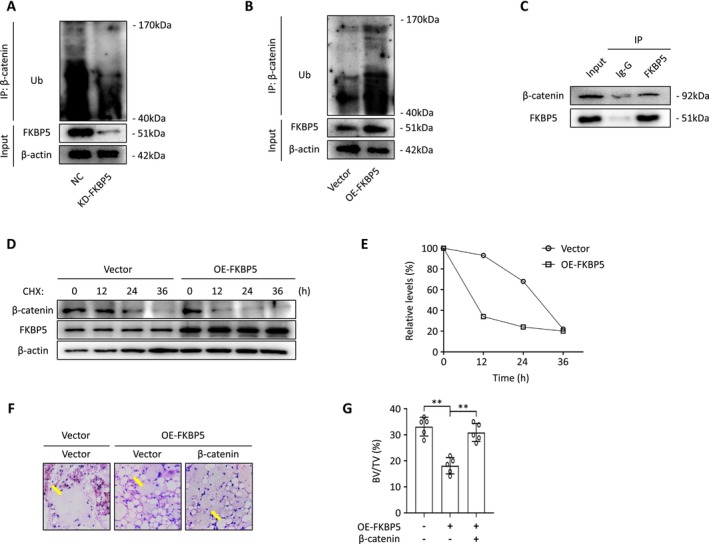
FKBP5 is essential for β‐catenin stabilisation. (A, B) The ubiquitination level of β‐catenin was enhanced by upregulating FKBP5 expression, and reduced by downregulating FKBP5 expression; (C) The co‐immunoprecipitation test showed the physical binding of FKBP5 and β‐catenin in human BMSCs; (D, E) BMSCs were treated with cycloheximide (CHX), a protein synthesis inhibitor, and the stability of β‐catenin was measured; (F, G) Representative H&E staining images and quantitative assessment of the bone tissue of transplants from different groups (yellow arrows represent newly formed bone).

**FIGURE 8:**
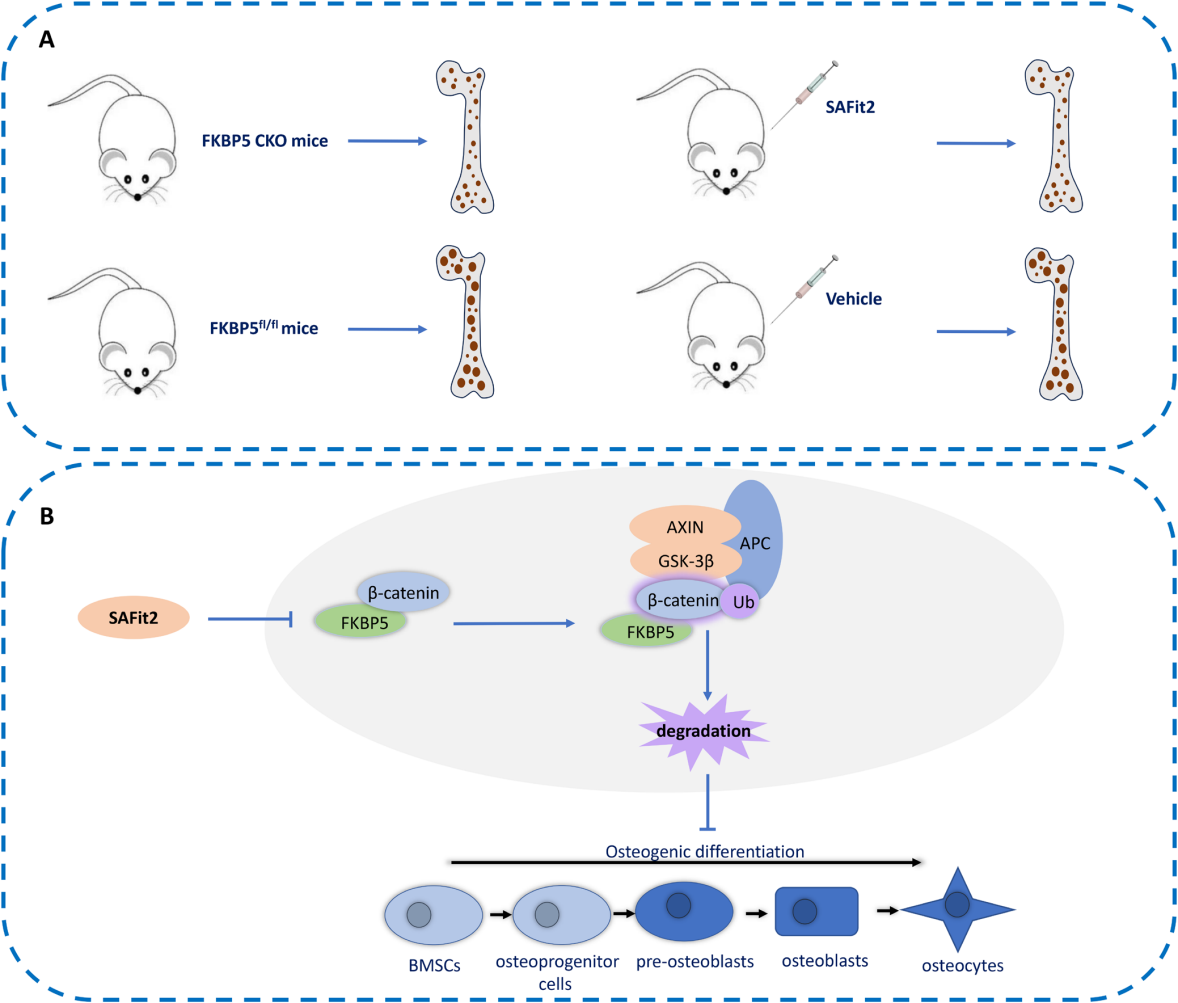
Graphical abstract of the study. (A) FKBP5 is associated with senile osteoporosis. Specifical deletion of FKBP5 in BMSCs alleviate senile osteoporosis in aged mice. Administration of SAFit2 enhances bone density in a senile osteoporosis model. (B) BMSC osteogenic differentiation is regulated by FKBP5 via the canonical WNT/β‐catenin signalling pathway.

Given the changes in β‐catenin protein levels caused by FKBP5 manipulation, we posited that the ubiquitin‐proteasome system plays a significant role in regulating Wnt signalling. Co‐immunoprecipitation assays confirmed the physical interaction between FKBP5 and β‐catenin in human BMSCs (Figure 7C). The ubiquitination of β‐catenin was evaluated after treatment with the proteasome inhibitor MG132 for eight hours. Results indicated that FKBP5 downregulation reduced β‐catenin ubiquitination, while FKBP5 overexpression increased it (Figure 7A,B). The stability of β‐catenin was also assessed when BMSCs were exposed to the protein synthesis inhibitor, cycloheximide (CHX). We observed that FKBP5 upregulation significantly shortened the half‐life of β‐catenin (Figure 7D,E). Additionally, we transfected FKBP5‐upregulated BMSCs with β‐catenin lentivirus or a control lentivirus. When these cells were implanted into muscle pockets on the hind limbs of nude mice, overexpression of β‐catenin restored the osteogenic differentiation ability in FKBP5‐upregulated BMSCs (Figure 7F,G). Finally, we illustrated the regulatory network of FKBP5 in BMSC osteogenic differentiation in Figure 8.

## 3 Discussion

As individuals age, the delicate balance between bone formation by osteoblasts and bone resorption by osteoclasts deteriorates, leading to dysfunctional bone remodelling [21, 22, 23]. This dysfunction contributes to increased fracture risk, impaired bone repair, and the onset of osteoporosis, a chronic metabolic disorder linked to ageing [24, 25]. Under various conditions and in response to different cytokines, BMSCs can differentiate into osteoblasts, adipocytes, or chondrocytes [26, 27]. Recent studies are shedding light on how maintaining metabolic balance in bones is influenced by the processes of osteogenic and adipogenic differentiation [28, 29].

FKBP5 has been extensively investigated in the context of neurological disorders, such as post‐traumatic stress disorder (PTSD), Alzheimer's disease (AD), and Parkinson's disease (PD) [18, 30, 31, 32, 33]. Furthermore, FKBP5 has been associated with ageing‐related changes, including increased cardiovascular risk and heightened inflammation [19, 34, 35, 36]. However, its specific contributions and mechanisms of action in relation to senile osteoporosis—a critical age‐related metabolic disease—remain poorly understood.

Our study revealed that BMSCs from elderly individuals exhibited several ageing‐related characteristics compared to those from younger individuals. These included a decreased rate of cell proliferation, elevated β‐galactosidase staining, increased expression levels of the age marker p21, and cell cycle arrest in the G1 phase [37, 38, 39]. Notably, FKBP5 expression increased concurrently with these changes, while expression levels in osteoclasts remained relatively stable. Functional analysis showed a strong correlation between FKBP5 expression and the osteogenic differentiation potential of BMSCs in elderly individuals. Specifically, downregulation of FKBP5 resulted in increased expression of osteogenic markers at both the mRNA and protein levels, along with enhanced ALP and alizarin red staining. Transplantation experiments further indicated that BMSCs with reduced FKBP5 expression displayed greater potential for osteogenic differentiation. In contrast, elevated FKBP5 levels were linked to decreased expression of osteogenic markers and impaired differentiation capacity, as demonstrated by lower ALP activity and mineralisation outcomes. Similar results were observed in mouse models, where targeted deletion of FKBP5 in BMSCs led to an osteoporotic phenotype characterised by reduced bone volume and trabecular number, as confirmed by micro‐CT analysis. These findings emphasise the critical role of FKBP5 in regulating osteogenic differentiation in BMSCs.

The osteogenic differentiation of BMSCs is significantly influenced by the canonical Wnt/β‐catenin signalling pathway [28, 40, 41]. Our results indicate that FKBP5 modulates this process by binding to β‐catenin, promoting its ubiquitination and subsequent degradation. This conclusion is supported by our detection of key signalling proteins and downstream gene expressions, along with luciferase assays.

Scientific studies aim to provide insights that can be translated into clinical applications. Given our demonstration of FKBP5's essential role in bone metabolism, we propose that FKBP5 may serve as a valuable biomarker for identifying and managing osteoporosis. Our evaluation of FKBP5 expression in BMSCs revealed a negative correlation with BMD and CT values in patients, underscoring its potential clinical relevance.

To explore the therapeutic implications of FKBP5 inhibition in osteoporosis, we administered SAFit2, a selective inhibitor of FKBP5 [42, 43]. SAFit2 is a promising compound that modulates stress responses by selectively targeting FKBP5, without affecting the closely related FKBP4 [44, 45, 46]. The dose of SAFit2 was selected with reference to previous studies to support its efficacy and safety [47, 48]. In our animal model of senile osteoporosis, SAFit2 administration resulted in improved BMD, suggesting that FKBP5 plays a pivotal role in osteoporosis management.

In conclusion, our study finds that FKBP5 expression in BMSCs increases with age, with levels inversely correlated to BMD and CT values in patients. Functional investigations affirm FKBP5's regulatory role in osteogenic differentiation, mediated through the canonical Wnt/β‐catenin signalling pathway, where FKBP5 promotes the ubiquitination and degradation of β‐catenin. Furthermore, administration of SAFit2 enhances BMD in a senile osteoporosis model, highlighting FKBP5 as a novel target for osteoporosis treatment. These findings offer a new perspective on therapeutic strategies for osteoporosis, emphasising the need for further exploration of FKBP5 in clinical settings.

## 4 Materials and Methods

### 4.1 Cell Culture

Human BMSCs were obtained from healthy donors, categorised as young (ages 18–37) and old (ages 66–93). The cells were cultured in α‐modified essential medium (α‐MEM; Sigma‐Aldrich, Missouri, USA) supplemented with 100 μg/mL penicillin–streptomycin sulfate (Sigma‐Aldrich, Missouri, USA) and 10% fetal bovine serum (FBS; Gibco, California, USA). The cultures were maintained in a humidified incubator at 37°C with 5% CO_2_.

### 4.2 In Vitro Differentiation Assays

BMSCs were seeded in 24‐well plates and induced to differentiate using adipogenic or osteogenic differentiation mediums (Cyagen, Guangzhou, China) as per the manufacturer's instructions. The differentiation media were refreshed every two days. Adipogenic differentiation was assessed through western blotting, quantitative RT‐PCR, and Oil Red O staining, while osteogenic differentiation was evaluated using western blotting, quantitative RT‐PCR, Alizarin Red staining, ALP staining, and an ALP activity assay.

### 4.3 RNA Isolation and Quantitative RT‐PCR Assays

Total RNA was isolated using TRIzol Reagent (Invitrogen, Carlsbad, CA, USA) according to the manufacturer's protocol. The RNA was quantified using a NanoDrop 2000 (Thermo, Waltham, USA) and reverse‐transcribed into complementary DNA (cDNA) using the PrimeScript RT Reagent Kit (TaKaRa, Shiga, Japan). Real‐time PCR was conducted on an ABI HT7900 (Applied Biosystems, Australia), and gene expression levels were normalised to β‐actin.

### 4.4 Western Blot Analysis and co‐Immunoprecipitation

Protein lysates were prepared using a Cell Lysis Buffer, with a Protease Inhibitor (Boster, Wuhan, China) added to prevent degradation. Protein concentrations were determined using the BCA Protein Assay Kit (Thermo Fisher Scientific). Proteins were separated via 10% SDS‐PAGE at 130 V for 80 min, followed by electroblotting onto membranes at 260 mA for 80 min. Membranes were blocked for 70 min and incubated overnight with primary antibodies at 4°C. After washing, secondary antibodies were applied for one hour at room temperature. Protein expression was measured using ImageQuant LAS 4000 (GE Healthcare).

For co‐immunoprecipitation, an immunoprecipitation kit from Abcam was utilised. Cells were lysed on ice for 20 min with a Lysis Buffer containing a Protease Inhibitor Cocktail. After centrifugation at 1000 g for 10 min, the supernatant was incubated overnight at 4°C with primary antibody. Protein A/G was added for an additional hour at 4°C. The beads were washed three times with Wash Buffer and analysed by western blot.

### 4.5 In Vitro Ubiquitination Assay

Cell Lysis Buffer was used to lyse cells treated with the proteasome inhibitor MG132 for eight hours. The lysate was incubated with specific antibodies for three hours at 4°C, followed by the addition of protein A/G Sepharose beads, which were rotated gently overnight at 4°C. The beads were collected by low‐speed centrifugation and washed three times with Wash Buffer. Immunoprecipitated proteins were eluted using SDS‐loading buffer and heated at 95°C for 5 min prior to western blot analysis.

### 4.6 Animal Models

All animal experiments were approved by the Animal Committee of Nanjing Medical University. Prx1‐cre mice and FKBP5flox/flox mice were obtained from Cyagen Bioscience (Guangzhou, China). FKBP5 was specifically deleted in BMSCs through interbreeding FKBP5flox/flox mice with Prx1‐cre transgenic mice. Mice were housed under standard conditions with unlimited access to food and water, maintained on a 12‐h light–dark cycle at room temperature.

### 4.7 BMSC Transplantation Experiment

This study involved eight‐week‐old immunocompromised mice, with all procedures approved by the First Affiliated Hospital Ethics Committee of Nanjing Medical University. Beta‐tricalcium phosphate (β‐TCP; Bio‐Lu, Shanghai, China) was used as a scaffold for BMSC transplantation into muscle pockets in the hind limbs. Mice were euthanised after eight weeks post‐transplantation under anaesthesia, and the transplants were harvested and preserved in 10% formalin. Samples were then decalcified with 10% EDTA, embedded in paraffin, sectioned using a Lecia RM2235 microtome (Leica, Heidelberg, Germany), and analysed with a BIOQUANT OSTEO imaging system (BIOQUANT, Nashville, TN, USA).

### 4.8 Plasmid Transfection

FKBP5 plasmid and control plasmid were constructed by RiboBio (Guangzhou, China), FKBP5 overexpression vector and empty vector were constructed by GenePharma (Shanghai, China). For virus production, 293 T cells were transfected with 12 μg expression plasmids or corresponding control, 3.6 μg envelope plasmid, and 9 μg packaging plasmid using Lipofectamine 3000. Viral supernatants were harvested at 48 h post‐transfection and filtered through 0.45 μm membranes (Sigma‐Aldrich, USA). BMSCs were transduced using viral particles with 6 μg/mL polybrene (Sigma‐Aldrich, USA).

### 4.9 Statistical Analysis

Statistical analyses were conducted using SPSS version 16.0 (IBM Corporation, New York, USA). All experiments were performed in triplicate to ensure reproducibility. Comparisons between two groups were made using the independent sample t‐test, while comparisons among multiple groups utilised one‐way analysis of variance (ANOVA). Data are presented as means ± standard deviation (SD), with statistical significance defined as *p* < 0.05.

## Author Contributions

**Bin Zhu:** conceptualization (equal), formal analysis (equal), funding acquisition (equal), methodology (equal), project administration (equal), writing – original draft (equal), writing – review and editing (equal). **Bowen Cai:** data curation (equal), formal analysis (equal), investigation (equal), methodology (equal), software (equal), writing – original draft (equal), writing – review and editing (equal). **Kaixiao Xue:** data curation (equal), investigation (equal), software (equal), writing – original draft (equal). **Shumin Zhou:** resources (equal), software (equal), writing – review and editing (equal). **Guoyong Yin:** conceptualization (equal), project administration (equal), supervision (equal), writing – review and editing (equal). **Jiahu Fang:** conceptualization (equal), visualization (equal), writing – review and editing (equal).

## Consent

The authors have nothing to report.

## Conflicts of Interest

The authors declare no conflicts of interest.

## Supporting information


Table S1.

## Data Availability

The data that support the findings of this study are available from the corresponding author upon reasonable request.
