# Supplementary material for: FKBP5 Induces Senescence in BMSCs and Inhibits Osteogenic Differentiation Through the Canonical WNT/β‐Catenin Signalling Pathway in Senile Osteoporosis
Source: J Cell Mol Med. 2025 Apr 20;29(8):e70552. doi: 10.1111/jcmm.70552 (PMC12009754; doi:10.1111/jcmm.70552)
Supplement: Supplementary file 1 — Table S1. [file JCMM-29-e70552-s001.docx]

Table S1. Baseline data of mice.

| Item | young mice | old mice | p value |
| --- | --- | --- | --- |
| Body weight (g) | 30.88±1.87 | 31.62±3.94 | 0.76 |
| BMD (g/cm^3^) | 0.22±0.019 | 0.18±0.0173 | 0.026 |
| BV/TV (%) | 25.07±1.65 | 21.07±1.48 | 0.023 |
| Th.Th (um) | 14.48±0.59 | 12.49±1.03 | 0.046 |
| Tb.Sp (um) | 39.81±2.36 | 44.62±1.23 | 0.018 |
| Tb.N (mm^-1^) | 9.39±0.84 | 7.59±0.53 | 0.011 |

BMD, bone mineral density; Tb.Sp, trabecular separation; BV/TV, bone volume/total volume; Tb.Th, trabecular thickness; Tb.N, trabecular number.
